# High Mobility Group Box Protein 1 Boosts Endothelial Albumin Transcytosis through the RAGE/Src/Caveolin-1 Pathway

**DOI:** 10.1038/srep32180

**Published:** 2016-08-30

**Authors:** Dan Shang, Tao Peng, Shanmiao Gou, Yiqing Li, Heshui Wu, Chunyou Wang, Zhiyong Yang

**Affiliations:** 1 Department of Vascular Surgery, Union Hospital, Tongji Medical College, Huazhong University of Science and Technology, 1277 Jiefang Avenue, Wuhan, Hubei Province 430022, China; 2 Department of Pancreatic surgery, Union Hospital, Tongji Medical College, Huazhong University of Science and Technology, 1277 Jiefang Avenue, Wuhan, Hubei Province 430022, China

## Abstract

High-mobility group box protein 1 (HMGB1), an inflammatory mediator, has been reported to destroy cell-cell junctions, resulting in vascular endothelial hyperpermeability. Here, we report that HMGB1 increases the endothelial transcytosis of albumin. In mouse lung vascular endothelial cells (MLVECs), HMGB1 at a concentration of 500 ng/ml or less did not harm cell-cell junctions but rapidly induced endothelial hyperpermeability to ^125^I-albumin. HMGB1 induced an increase in ^125^I-albumin and AlexaFluor 488-labeled albumin internalization in endocytosis assays. Depletion of receptor for advanced glycation end products (RAGE), but not TLR2 or TLR4, suppressed HMGB1-induced albumin transcytosis and endocytosis. Genetic and pharmacological destruction of lipid rafts significantly inhibited HMGB1-induced albumin endocytosis and transcytosis. HMGB1 induced the rapid phosphorylation of caveolin (Cav)-1 and Src. Either RAGE gene silencing or soluble RAGE suppressed Cav-1 Tyr14 phosphorylation and Src Tyr418 phosphorylation. The Src inhibitor 4-amino-5-(4-chlorophenyl)-7-(*t*-butyl) pyrazolo[3,4-*d*] pyrimidine (PP2) blocked HMGB1-induced Cav-1 Tyr14 phosphorylation. PP2 and overexpression of Cav-1 with a T14F mutation significantly inhibited HMGB1-induced transcytosis and albumin endocytosis. Our findings suggest that HMGB1 induces the transcytosis of albumin via RAGE-dependent Src phosphorylation and Cav-1 phosphorylation. These studies revealed a new mechanism of HMGB1-induced endothelial hyperpermeability.

High mobility group box 1 (HMGB1), a member of the high mobility group (HMG) protein family named after its fast migration property in PAGE, is a non-histone chromosomal protein that is widespread in eukaryotic cells^1^. Structurally, HMGB1 is composed of a 215 amino acid single-chain polypeptide and has a molecular weight of approximately 25 kDa. It is highly conserved and contains an N-terminus with a basic (positively charged) lysine residue and an acidic (negatively charged) C-terminus with a high amount of aspartic and glutamic acid residues^1^.

HMGB1 has been formerly known for its intracellular functions in contributing to the stabilization of the nucleosome and bent DNA formation as well as repair^2^. Recent studies have shown that HMGB1 is also involved in the control of mitochondrial quality and the regulation of cell autophagy^3^,^4^. HMGB1 can either be passively released from necrotic cells or secreted actively by a variety of cells into the extracellular environment. Extracellular HMGB1 acts as a pro-inflammatory cytokine in the inflammatory response and attracts stem cells to home into areas of inflammation, promoting the regeneration process^5^. In addition, it is considered to be associated with the growth and proliferation of multiple types of tumours^6^.

In the past, for the first time, it was found that HMGB1 was released into the extracellular environment as an important late pro-inflammatory cytokine during sepsis^7^. Since then, a large number of studies have shown that HMGB1 is involved in the progression of burns, severe acute pancreatitis, haemorrhagic shock, disseminated intravascular coagulation, rheumatoid arthritis, systemic lupus erythaematosus, and other diseases^5^,^8^. Early studies have demonstrated that HMGB1 exhibits typical pro-inflammatory cytokine activity and activates inflammatory cells (e.g., monocytes or macrophages, neutrophils, vascular endothelial cells) to release cytokines and chemokines (e.g., tumour necrosis factors and interleukins)^5^,^8^,^9^,^10^. The main receptors of HMGB1 include receptor for advanced glycation end products (RAGE), TLR4 and TLR2^5^.

A severe systemic inflammatory response can cause endothelial injury and high capillary permeability, in turn resulting in severe capillary leakage during the initial stages of extensive burns, severe acute pancreatitis, and severe sepsis^11^,^12^. High capillary permeability is associated with water, electrolyte, and albumin leakage into the tissue interspace. Albumin leakage occurs through two pathways, i.e., the paracellular pathway and transcytosis^13^. Albumin leakage via the paracellular pathway is a result of damage to cell-cell junctions^13^, whereas albumin transcytosis results from endocytosis after binding with the receptor gp60 and subsequent basolateral excretion^14^. Albumin endocytosis occurs via the caveolin (Cav)-1-dependent endocytic pathway^14^. In the absence of Cav-1, vascular endothelial cells can transfer albumin through the clathrin-dependent endocytic pathway^15^.

It has been reported that high concentrations of HMGB1 (≥5 μg/mL), resulting in vascular barrier damage, quickly destroy the junctions between pulmonary vascular endothelial cells^16^, whereas lower concentrations of HMGB1 (200 ng/mL) for less than 12 h caused no destruction of the cell-cell junctions^16^,^17^. Several studies showed that although the HMGB1 level was elevated in patients with severe acute pancreatitis or sepsis, it remained far less than 200 ng/mL^18^,^19^,^20^. It was observed that exposure to lower concentrations (<200 ng/ml) of HMGB1 for a shorter time period could not affect the integrity of endothelial cell-cell junctions. However, the effect of HMGB1 on albumin transcytosis remains unclear.

In vascular endothelial cells, Cav-1 phosphorylation by Src (the signalling protein associated with Cav-1) can lead to the increased transcellular transport of albumin^21^. In addition, after binding with its ligand, RAGE activates Src and downstream signals^22^. We speculated that HMGB1 induces endothelial albumin transcytosis through Src activation. In the present study, the results showed that a lower concentration (100 ng/ml) of HMGB1 did not damage the integrity of the junctions between mouse lung vascular endothelial cells (MLVECs) but boosted albumin transcytosis via RAGE-dependent Src and Cav-1 phosphorylation, resulting in vascular endothelial hyperpermeability to albumin.

## Results

### HMGB1 induced pulmonary vascular endothelial hyperpermeability to albumin

**Figure 1:**
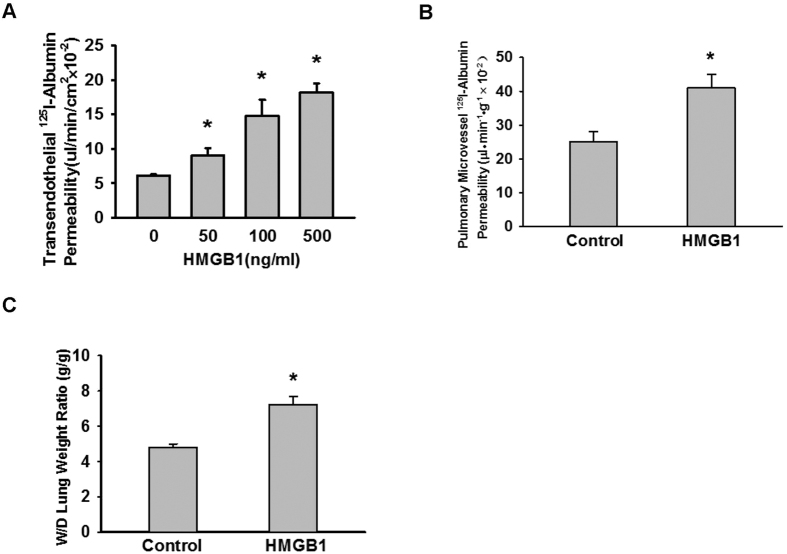
HMGB1 induced pulmonary vascular endothelial hyperpermeability to albumin. (**A**) HMGB1 induced albumin hyperpermeability of MLVEC monolayers. ^125^I-albumin and different concentrations of HMGB1 (0, 50, 100, 500 ng/mL) were added into the upper chamber of a Transwell chamber containing MLVEC monolayers. After 1 h of incubation, 200 μL of liquid sample was removed from the lower chamber for radioactivity detection (n = 4 to 6 for each group). (**B,C**) HMGB1 induced mouse pulmonary vascular hyperpermeability to albumin (**B**) and pulmonary oedema (**C**). The mouse lung samples were irrigated with a solution containing 0 or 100 ng/mL HMGB1 and ^125^I-albumin via pulmonary artery perfusion. After 30 min, the lungs were perfused with Krebs solution, weighed and used for radioactivity detection (n = 6/each group). After oven-drying at 60 °C for 72 h, the final weight was measured to calculate the wet/dry (W/D) weight ratio. *Compared with the control group, *p* < 0.05.

We examined the effects of HMGB1 on the permeability of the pulmonary vascular endothelium using a Transwell chamber assay. After a 1-h treatment of MLVEC monolayers with 0, 50, 100, and 500 ng/mL HMGB1 and ^125^I-albumin, the radioactivity of the liquid in the lower chamber was tested. We found that the permeability of MLVEC monolayers to ^125^I-albumin increased with increasing concentrations of HMGB1 (Fig. 1A). To verify the role of HMGB1 at the organ level, we performed the experiment with mouse lungs that were treated via pulmonary vascular perfusion with HMGB1 (100 ng/mL) and ^125^I-albumin for 30 min. We found that the lung W/D weight ratio (Fig. 1B) and the extravascular ^125^I-albumin level (Fig. 1C) were significantly higher in the HMGB1 group than in the control group. These results suggested that lower concentrations of HMGB1 resulted in the hyperpermeability of the pulmonary vascular endothelium to albumin as well as pulmonary oedema.

### Lower concentrations of HMGB1 did not damage the endothelial barrier

Albumin leakage can occur through the paracellular pathway or through transcytosis^13^. Studies have shown that HMGB1 stimulation destroys the integrity of endothelial cell junctions either at high concentrations or when exposed to cells for a longer period of time^16^,^17^.

**Figure 2:**
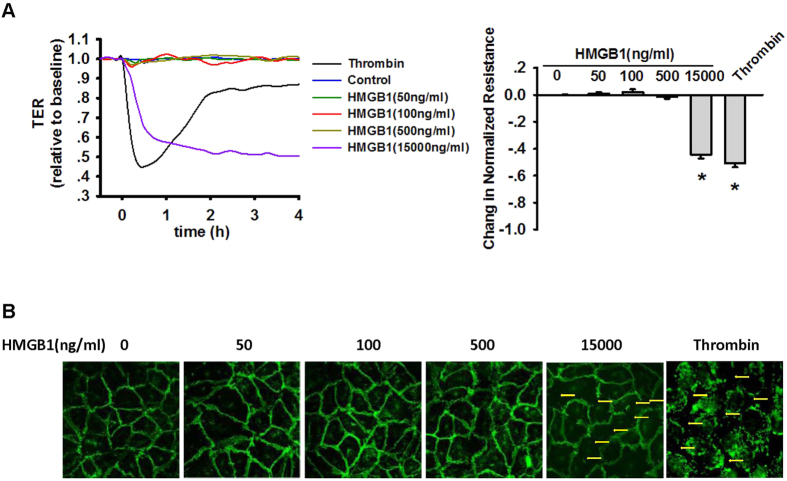
Low concentrations of HMGB1 do not affect the barrier function of endothelial cells. (**A**) Effect of HMGB1 on the TER in MLVEC monolayers. MLVEC monolayers were treated using 0, 50, 100, and 500 ng/mL HMGB1, with thrombin and 15 μg/mL HMGB1 as positive controls. Quantitative analysis of the data at 60 min after the treatment is shown on the right. (**B**) After MLVEC monolayers were treated with 0, 50, 100, 500 and 15000 ng/mL HMGB1 and Thrombin for 30 min, indirect immunofluorescence staining of VE-cadherin showed no continuous interruption of fluorescence or formation of intercellular gaps (yellow arrows) except in the positive control. For each group, n = 4 to 6. *Compared with the control group, *p* < 0.05.

The integrity of the cell-cell junctions was measured to further clarify the mechanism through which lower concentrations of HMGB1 affect the albumin hyperpermeability of the vascular endothelium. MLVEC monolayers were treated using 0, 50, 100 and 500 ng/mL HMGB1, with thrombin and 15 μg/mL HMGB1 as positive controls. The resulting changes in TER were evaluated, and the results showed that the TER in MLVEC monolayers rapidly decreased after thrombin administration but remained unchanged after HMGB1 stimulation (Fig. 2A).

Indirect immunofluorescence staining of VE-Cadherin was used to confirm the changes in cell-cell junctions, and 15 μg/mL HMGB1 and Thrombin was used as a positive control. Addition of 0–500 ng/mL HMGB1 caused no increase in the intercellular gaps between MLVECs (Fig. 2B). Collectively, these results indicated that 0–500 ng/mL HMGB1 caused no rapid destruction of the cell-to-cell junctions of MLVECs.

### HMGB1 increased the endothelial transcytosis of albumin

**Figure 3:**
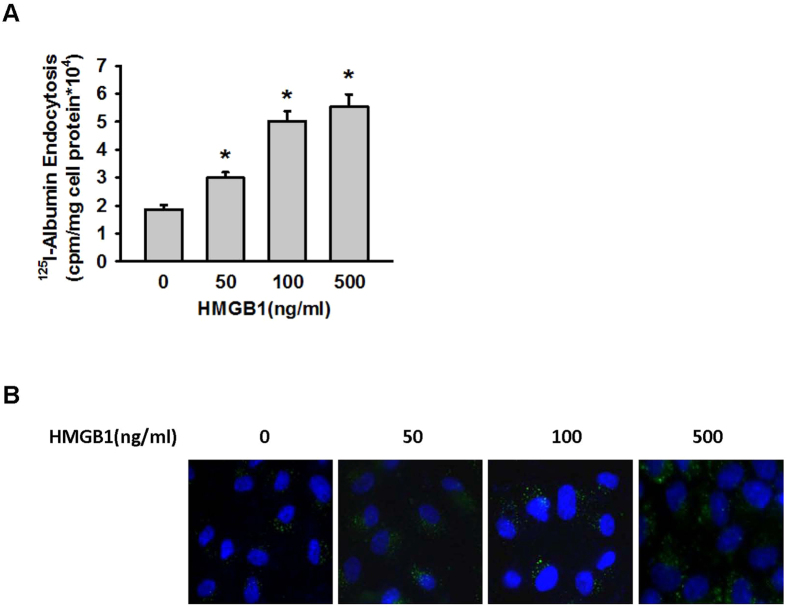
HMGB1 induced albumin endocytosis by vascular endothelial cells. (**A**) MLVEC monolayers were treated with HMGB1 and ^125^I-albumin. After 30 min, the cells were lysed for radioactivity detection. *Compared with the control group, *p* < 0.05. (**B**) MLVEC monolayers were treated with HMGB1 and AlexaFluor 488-labeled albumin. After 30 min, the cells were examined by confocal microscopy to quantify the amount of albumin endocytosed. For each group, n = 4 to 6.

Albumin endocytosis was assayed to evaluate whether HMGB1 results in increased transcytosis of albumin by vascular endothelial cells. Endothelial cells were stimulated with HMGB1 (0, 50, 100, and 500 ng/mL) and ^125^I-albumin. ^125^I-albumin endocytosis by MLVEC monolayers increased with increasing concentrations of HMGB1 (Fig. 3A). AlexaFluor 488-labeled albumin was also used in the endocytosis assay. Under a confocal microscope, green fluorescence in MLVECs increased with the increase of HMGB1 concentration (Fig. 3B). Combined with the results of the Transwell-based ^125^I-albumin permeability assay (Fig. 1A), these data suggested that increasing concentrations of HMGB1 caused albumin hyperpermeability by inducing the transcytosis of albumin in vascular endothelial cells.

### HMGB1-induced endothelial transcytosis of albumin was dependent on RAGE but not on TLR2/4

**Figure 4:**
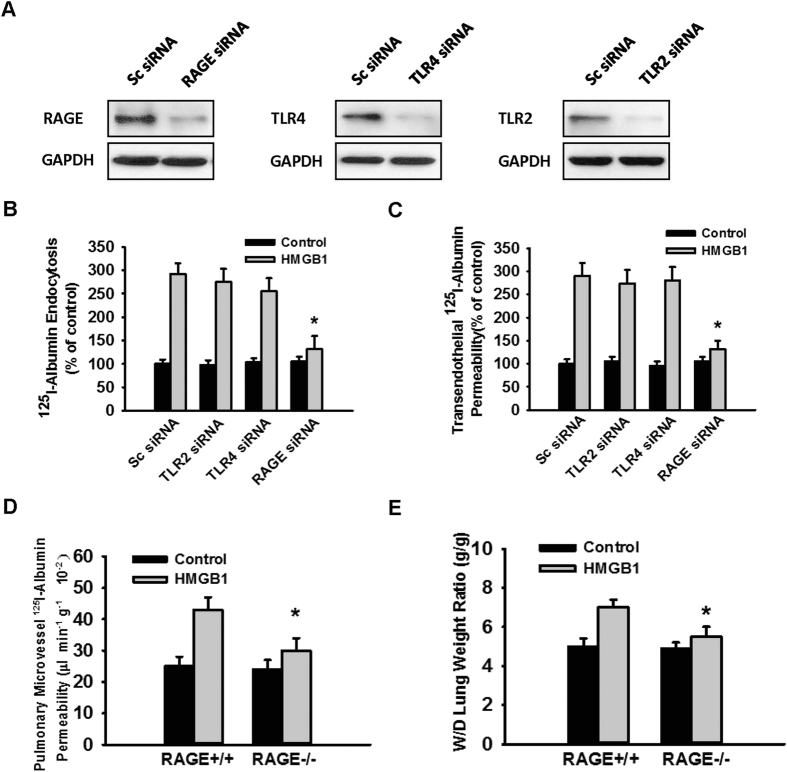
RAGE mediated the HMGB1-induced transcytosis of albumin. (**A**) RAGE, TLR2, and TLR4 expression was silenced successfully in MLVECs. The efficiency of gene silencing was tested by immunoblotting assay. (**B,C**) RAGE gene silencing decreased the HMGB1-induced endothelial transcytosis of albumin. After silencing the expression of the target gene, MLVECs were grown in 6-well plates (**B**) or in Transwell chambers (**C**). When the cells formed confluent monolayers, 0 or 100 ng/mL HMGB1 and ^125^I-albumin were added for assays of albumin endocytosis (**B**) and transendothelial permeability (**C**). (**D,E**) The RAGE^+/+^ and RAGE^−/−^ mouse lung samples were irrigated with a solution containing 0 or 100 ng/mL HMGB1 and ^125^I-albumin via pulmonary artery perfusion. After 30 min, the lungs were perfused with Krebs solution, weighed and used for radioactivity detection (n = 6/each group). Then, the lungs were oven-dried. *Compared with the HMGB1-treated RAGE^+/+^ group, *p* < 0.05.

At the surface of vascular endothelial cells, the three main receptors of HMGB1 are RAGE, TLR2, and TLR4^5^. After silencing of these three genes (Fig. 4A), we observed that only *RAGE* depletion blocked HMGB1-induced endocytosis and albumin transcytosis (Fig. 4B,C). To verify the role of RAGE on HMGB1-induced albumin transcytosis at the organ level, we evaluated the lungs of RAGE^−/−^ and RAGE^+/+^ mice after pulmonary vascular perfusion with HMGB1 and ^125^I-albumin for 30 min. We found that the extravascular ^125^I-albumin level (Fig. 4D) and the W/D weight ratio (Fig. 4E) was lower in RAGE^−/−^ lungs. Thus, it can be concluded that HMGB1-induced albumin transcytosis was dependent on RAGE but not on TLR2/4.

### HMGB1 induced Cav-1-dependent endothelial transcytosis of albumin

**Figure 5:**
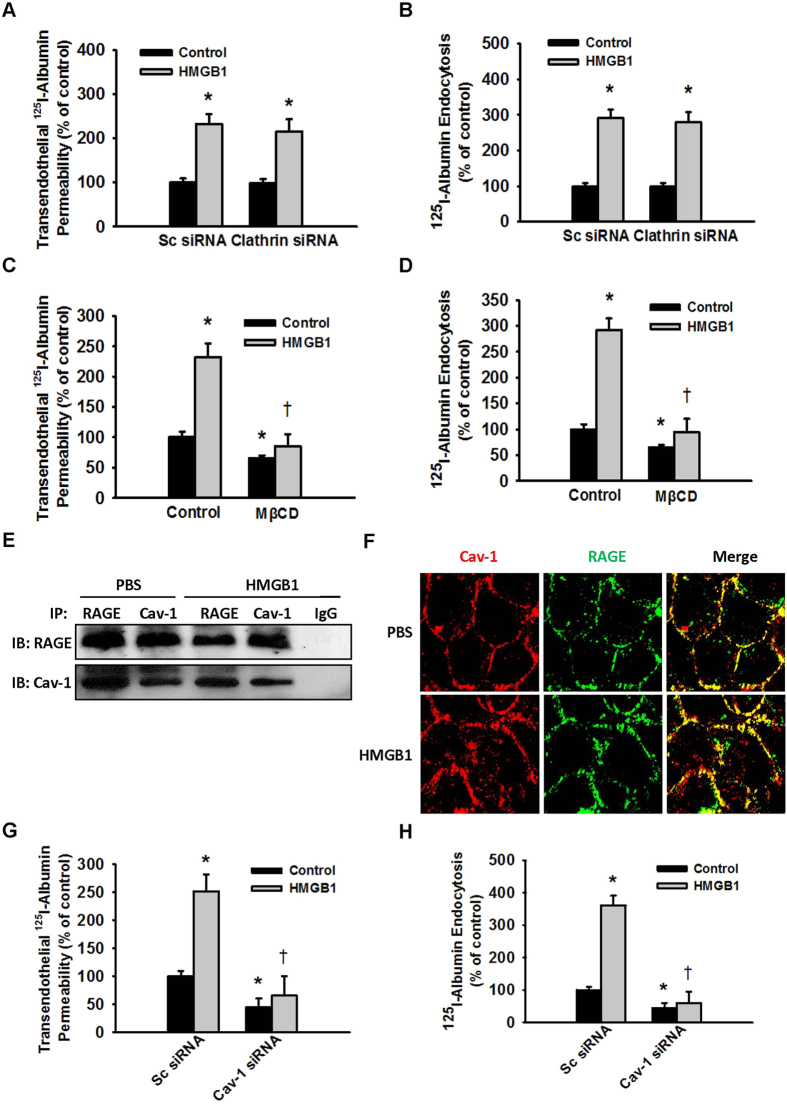
HMGB1 induced the Cav-1-dependent endothelial transcytosis of albumin. (**A,B**) Effect of MβCD on the HMGB1-induced endocytosis and transcytosis of albumin. Prior to exposure to 100 ng/mL HMGB1, MLVECs were pretreated with 2 mM MβCD or DMSO for 30 min. (**C,D**) Effect of clathrin knockdown on the HMGB1-induced endocytosis and transcytosis of albumin. (**E**) Co-IP results showed the binding of Cav-1 and RAGE in MLVECs. MLVECs were cultured with or without 100 ng/mL HMGB1 for 1 h. (**F**) Co-localization of Cav-1 with RAGE on the cell membrane of MLVECs. MLVECs were cultured with or without 100 ng/mL HMGB1 for 1 h. Double staining of Cav-1 (red) and RAGE (green) in MLVECs was performed using indirect immunofluorescence. (**G,H**) Effect of Cav-1 knockdown on the HMGB1-induced endocytosis and transcytosis of albumin. After clathrin expression (**C,D**) or Cav-1 expression (**G,H**) was silenced, MLVECs were grown in Transwell chambers (**C,G**) or 6-well plates (**D,H**). When the cells formed confluent monolayers, 0 or 100 ng/mL HMGB1 and ^125^I-albumin were added for assays of albumin endocytosis (**D,H**) and transendothelial permeability (**C,G**). n = 4 to 6 for each group. *Compared with the non-HMGB1-treated control (**A,B**) or the sc siRNA group (**C,D,G,H**), *p* < 0.05. ^†^Compared with the HMGB1-treated control (**A,B**) or the sc siRNA group (**C,D,G,H**), *p* < 0.05.

Endocytosis and transcytosis of albumin in vascular endothelial cells are thought to be Cav-1-dependent^14^, although studies have shown that in the absence of Cav-1, transport of albumin in the vascular endothelial cells takes place via the clathrin-dependent endocytic pathway^15^. To further clarify whether the albumin endocytic pathway affected by HMGB1 is dependent on Cav-1 or clathrin, we used methyl-β-cyclodextrin (MβCD) and clathrin depletion to block the Cav-1-dependent and clathrin-dependent endocytic pathways, respectively^23^. It was found that MβCD, rather than clathrin siRNA, blocked HMGB1-induced endocytosis and albumin transcytosis (Fig. 5A–D).

As described above, HMGB1 mediated the albumin hyperpermeability of endothelial cells via its receptor RAGE (Fig. 4A–E). We thus studied the positional relationship of RAGE and Cav-1 in the cell membrane. The results of co-immunoprecipitation and immunofluorescence staining showed that Cav-1 bound with RAGE on the membrane of MLVECs, and HMGB1 stimulation seemed not to alter their colocalization (Fig. 5E,F). Furthermore, we silenced the expression of Cav-1 in MLVECs and found that HMGB1-induced endocytosis and transcytosis of albumin was blocked (Fig. 5G,H). These data showed that HMGB1 induced the Cav-1-dependent endothelial transcytosis of albumin.

### HMGB1 induced the endothelial transcytosis of albumin via Cav-1 phosphorylation

**Figure 6:**
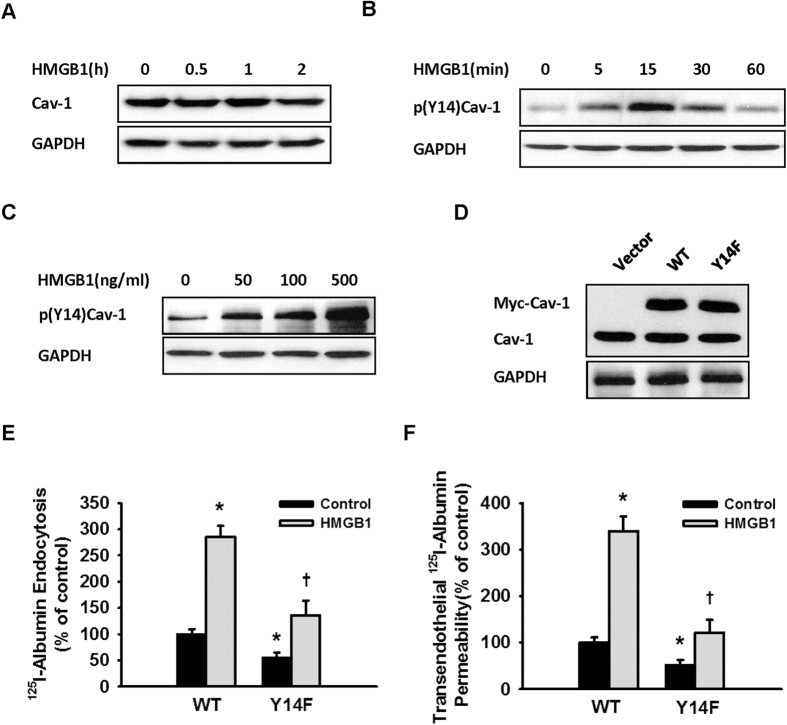
Cav-1 Y14 phosphorylation mediated HMGB1-induced albumin hyperpermeability. (**A**) HMGB1 did not affect Cav-1 expression. (**B**) Effect of HMGB1 on Cav-1 Y14 phosphorylation. MLVEC monolayers were exposed to 100 ng/mL HMGB1. The cells were lysed at the indicated times for immunoblotting assays of Cav-1 (**A**) and p(Y14)Cav-1 expression (**B**). (**C**) MLVEC monolayers were exposed to different concentrations of HMGB1 for 15 min. The cells were lysed for immunoblotting to evaluate p(Y14)Cav-1 expression. (**D**) Cav-1 Y14F and wild-type Cav-1 were overexpressed in MLVECs. The expression efficiency was detected by immunoblotting assay. All blots are representative of 3 separate experiments. Overexpression of a Y14 phosphorylation-defective Cav-1 mutant significantly inhibited HMGB1-induced ^125^I-albumin endocytosis (**E**) and transcellular transport (**F**). The concentration of HMGB1 used in the experiment was 100 ng/mL. (**E,F**) n = 4 to 6 for each group.

Induction of Cav-1 expression^24^ or phosphorylation^21^ can increase the transendothelial transport of albumin. A western blot assay showed that HMGB1 did not affect the Cav-1 expression level in MLVECs (Fig. 6A).

Cav-1 tyrosine phosphorylation (Y14) initiates the endocytosis and transcytosis of albumin^14^. The next experiment was based on the effects of HMGB1 on Cav-1 phosphorylation. HMGB1 was found to induce Cav-1 Y14 phosphorylation in MLVECs in a dose-dependent manner (Fig. 6B,C). Furthermore, overexpression of a Cav-1 Y14 phosphorylation-defective mutant (Cav-1 Y14F) in MLVECs (Fig. 6D) resulted in significantly reduced endocytosis and transcytosis of albumin compared to the wild-type group (Fig. 6E,F). These results indicated that Y14 phosphorylation of Cav-1 mediated the HMGB1-induced endothelial transcytosis of albumin.

### Src phosphorylation mediated the HMGB1-induced Cav-1 phosphorylation and endothelial transcytosis of albumin

**Figure 7:**
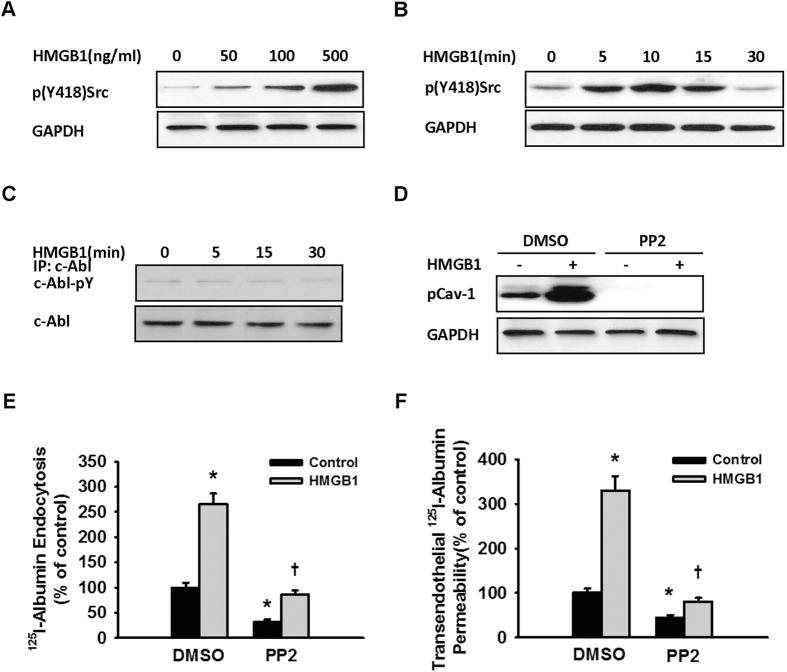
Src phosphorylation mediated HMGB1-induced Cav-1 phosphorylation and endothelial albumin transcytosis. (**A,B**) Effect of HMGB1 on Src Y418 phosphorylation. (**C**) Effect of HMGB1 on c-Abl phosphorylation. MLVEC monolayers were exposed to different concentrations of HMGB1 for 10 min and then lysed for immunoblotting assays of p(Y418)Src expression (**A**); MLVEC monolayers were exposed to 100 ng/mL HMGB1, and the cells were lysed at the indicated times for immunoblotting to evaluate p(Y418)Src expression (**B**); or anti-c-Abl specific antibody was used for immunoprecipitation, and anti-tyrosine phosphorylation antibody was then used for the detection of the protein phosphorylation level (**C**). (**D**) Effect of pp2 on HMGB1-induced Cav-1 Y14 phosphorylation. MLVEC monolayers were pretreated with 15 μM pp2 for 15 min and then treated with 100 ng/mL HMGB1 for 15 min. The cells were finally lysed for immunoblotting to evaluate p(Y14)Cav-1 expression. All blots are representative of 3 separate experiments. (**E,F**) pp2 inhibited HMGB1-induced ^125^I-albumin endocytosis and transcytosis. MLVEC monolayers were pretreated with 15 μM pp2 for 15 min and then treated with 100 ng/mL HMGB1 for 1 h (n = 4 to 6 for each group).

Cav-1 Y14 phosphorylation is catalysed by both Src, the major catalysing enzyme^14^ and c-Abl^21^,^25^. We measured the phosphorylation-mediated activation of Src in MLVECs after HMGB1 stimulation and found that Src was activated rapidly via phosphorylation in a dose-dependent manner by exposure to HMGB1 (Fig. 7A,B). However, the use of an inhibitor of Src activation, 4-amino-5-(4-chlorophenyl)-7-(*t*-butyl) pyrazolo[3,4-*d*] pyrimidine (PP2), blocked HMGB1-induced Cav-1 phosphorylation (Fig. 7D) and the transcytosis of albumin (Fig. 7E,F). In addition, after exposure to HMGB1, the activation of c-Abl via phosphorylation was not observed in MLVECs (Fig. 7C). Hence, HMGB1-induced Cav-1 phosphorylation and albumin transcytosis were dependent on Src phosphorylation.

### RAGE mediated HMGB1-induced Src phosphorylation and Cav-1 phosphorylation

**Figure 8:**
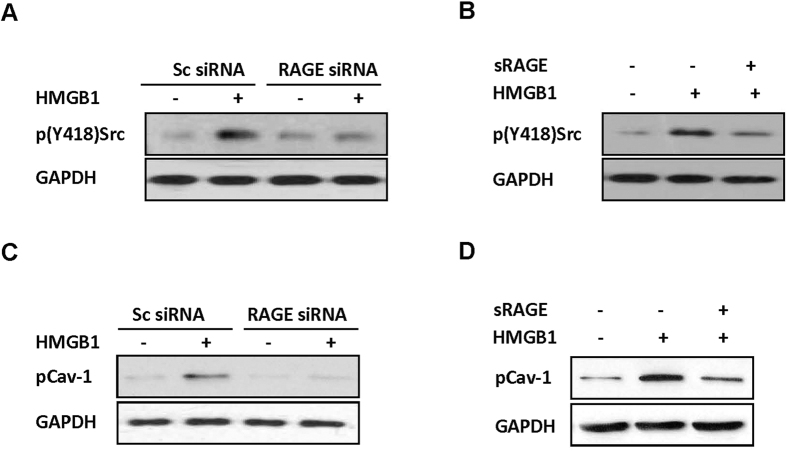
RAGE mediated HMGB1-induced Src and Cav-1 phosphorylation. *RAGE* gene silencing inhibited HMGB1-induced Src Y418 (**A**) and Cav-1 Y14 (**C**) phosphorylation. RAGE expression in MLVECs was silenced using siRNA, and the cells were then stimulated with 100 ng/mL HMGB1. After 10 (**A**) or 15 min (**C**), the cells were lysed to extract the proteins. sRAGE inhibited HMGB1-induced Src Y418 (**B**) and Cav-1 Y14 (**D**) phosphorylation. MLVEC monolayers were pretreated with sRAGE for 15 min, and the cells were then treated with 100 ng/mL HMGB1. After 10 (**B**) or 15 min (**D**), the cells were lysed for protein extraction. The p(Y418)Src and p(Y14)Cav-1 levels were detected by immunoblotting assays. n = 3/each group.

We next sought to further confirm the role of RAGE in HMGB1-induced Src phosphorylation and Cav-1 phosphorylation was studied. The results were consistent with the previous findings and confirmed that RAGE gene silencing (Fig. 8A,C) or sRAGE (Fig. 8B,D) blocked HMGB1-induced Src Y418 phosphorylation and Cav-1 Y14 phosphorylation.

### All-thiol HMGB1, but not disulfide HMGB1, induced the endothelial transcytosis of albumin

**Figure 9:**
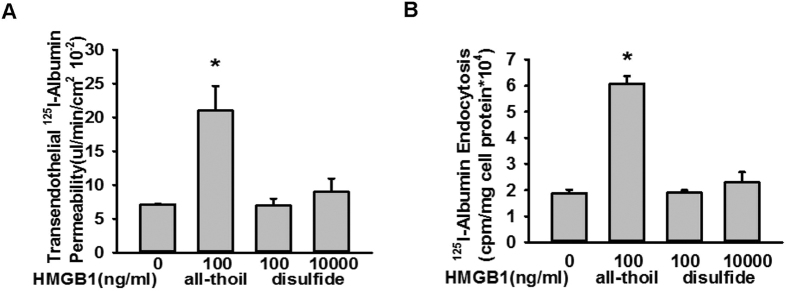
All-thiol HMGB1, but not disulfide HMGB1, enhanced the endocytosis and transcytosis of albumin. (**A**) ^125^I-albumin and HMGB1 were added into the upper chamber of a Transwell chamber cultured with MLVEC monolayers. After 1 h of incubation, 200 μL of liquid sample was removed from the lower chamber for radioactivity detection. (**B**) MLVEC monolayers were treated with HMGB1 and ^125^I-albumin. After 30 min, the cells were lysed for radioactivity detection. For each group, n = 4 to 6. *Compared with the non-HMGB1 treatment group, *p* < 0.05.

The redox state of HMGB1 modulates its extracellular functions^26^. All-thiol HMGB1 uses RAGE signalling, while disulfide HMGB1 recognizes TLR4. We found that all-thiol HMGB1, but not disulfide HMGB1, induced the endocytosis and transcytosis of albumin (Fig. 9A,B).

## Discussion

Our study is the first report on vascular endothelial hyperpermeability induced by pathophysiological concentrations of HMGB1. The results showed that 100 ng/mL of HMGB1 increased albumin transcytosis. Huang *et al.*^17^ found that there were no effects of HMGB1 (100 ng/mL) on the permeability of HUVEC monolayers because they did not study the effects of HMGB1 on the transcellular pathway. Similarly, Wolfson *et al.*^16^ only focused on the effects of HMGB1 on cell-cell junctions. In our study, TER and VE-Cadherin staining results showed that 100 ng/mL HMGB1 did not destroy the cell-cell junctions of MLVECs but still induced ^125^I-albumin hyperpermeability. As was confirmed in the endocytosis assays of ^125^I- and AlexaFluor 488-labeled albumins, this endothelial hyperpermeability was achieved by increasing the endocytosis and transcellular transport of albumin.

The three receptors of HMGB1 are RAGE, TLR4, and TLR2, among which RAGE seems to be altered when HMGB1 performs different functions in various cells. Animal experiments have shown that knockout of RAGE had a protective effect on mice with sepsis induced by caecal ligation puncture, and this protective effect was eliminated by exogenous RAGE expression in the endothelial cells and bone marrow cells of RAGE knockout mice^22^. This result suggested that RAGE is involved in the progression of sepsis in mice. RAGE knockout is more effective in reducing the HMGB1-induced release of pro-inflammatory cytokines than TLR2 knockout in bone marrow–derived macrophages, suggesting that RAGE is the main receptor of HMGB1 recognized by macrophages^22^. RAGE seems to be more important for the function of HMGB1 in vascular endothelial cells. Activation of HUVECs by HMGB1 can be inhibited by more than 50% with RAGE-neutralizing antibodies^9^. Huang *et al.*^17^ and Wolfson *et al.*^16^ showed that the damage caused by HMGB1 to cell-cell junctions was dependent on RAGE but was independent of TLR4 and TLR2. The present study showed that the HMGB1-induced transcytosis of albumin was dependent on RAGE but not TLR4 and TLR2. These data are consistent with reports that RAGE KO protected mice from acute lung injury^27^.

Albumin can be endocytosed through the clathrin-dependent and Cav-1-dependent pathways^15^. After MβCD or siRNA were used to destruct lipid rafts and to silence Cav-1 expression, respectively, we found that HMGB1-induced albumin endocytosis and transcytosis were significantly inhibited; however, clathrin knockdown did not block HMGB1-induced albumin endocytosis or transcytosis. These results indicated that HMGB1 increased vascular endothelial permeability to albumin by upregulating the Cav-1-dependent transcytosis of albumin.

Upregulated *Cav-1* expression allows increased endocytosis and transcytosis of albumin^24^. Our data showed that HMGB1 did not increase the expression level, but raised the phosphorylation level, of Cav-1 in endothelial cells. Phosphorylation of Cav-1 Tyr14 is considered to be a key step to initiate the endocytosis of albumin. A study has shown that hydrogen peroxide increased the transcytosis of albumin by inducing Cav-1 Tyr14 phosphorylation^21^. In the present study, HMGB1-induced Cav-1 Tyr14 phosphorylation was mediated by RAGE, as either RAGE gene silencing or sRAGE blocked Cav-1 Tyr14 phosphorylation. Overexpression of Cav-1 with a Tyr14 phosphorylation-defective mutant significantly inhibited the HMGB1-induced transcytosis and endocytosis of albumin, strongly suggesting that HMGB1 increases endothelial permeability by regulating Cav-1 Tyr14 phosphorylation. It has been reported that Cav-1 Tyr14 phosphorylation is regulated by Src and c-Abl^21^,^25^. We found that HMGB1 induced Src phosphorylation, but c-Abl was not activated by phosphorylation.

Redox modulates the extracellular functions of HMGB1^26^. All-thiol HMGB1 used RAGE signalling, while disulfide HMGB1 recognized TLR4. We found that all-thiol HMGB1, but not disulfide HMGB1, induced the endocytosis and transcytosis of albumin. This is consistent with our findings that RAGE mediates HMGB1-induced albumin transcytosis.

HMGB1 requires additional factors to exert its inflammatory activity, including LPS, IL-1, and nucleosomes^28^. In a real pathophysiological environment, HMGB1 co-exists with these inflammatory mediators. In the presence of these mediators, the effect of HMGB1 on vascular permeability may have additional features that need to be studied.

## Materials and Methods

### Materials

Recombinant HMGB1 was purchased from R&D Systems (Minneapolis, MN). All-thiol HMGB1 and disulfide HMGB1 were obtained from HMGBiotech. Protein A+G Agarose beads, HRP-conjugated secondary Abs, and anti-GAPDH, TLR4 and TLR2 Abs were from Santa Cruz Biotechnology. Anti-Cav-1, p(Y14)Cav-1, p(Y418)Src, RAGE, c-Abl, and phospho-tyrosine Abs, normal rabbit IgG and normal mouse IgG were from Cell Signalling Technology. TLR2, TLR4, RAGE, clathrin and Cav-1 siRNA, sc siRNA and Dharmafector 1 transfection reagent were obtained from Thermo Scientific. AlexaFluor 488-conjugated albumin was from Molecular Probes. TLR2- and TLR4-neutralizing Abs were from Abcam. Recombinant soluble RAGE (sRAGE) was from Biotrend. ^125^I-albumin was purchased from PerkinElmer, Inc. Amaxa basic nucleofector kits for primary endothelial cells were obtained from Lonza. RIPA buffer and ECL reagents were from Pierce Biotechnology. Bicinchoninic acid kits and sample buffer were from Bio-Rad. AlexaFluor 568- and AlexaFluor 488-conjugated secondary Abs were from Invitrogen. MEM D-Val medium and FBS were from Gibco. Biotin-conjugated rat anti-mouse CD31 (PECAM-1) monoclonal antibody and BD IMag Streptavidin Particles Plus-DM were from BD Pharmingen. All other reagents were obtained from Sigma unless otherwise specified.

### Animals

C57BL/6 mice weighing 25–30 g were obtained from the Experimental Animal Centre of Tongji Medical College, Huazhong University of Science and Technology (Wuhan, China). RAGE KO mice were generated on a C57BL/6 background^29^. The mice were housed under specific pathogen-free conditions, fed with autoclaved food, and used in experiments at 8–12 wks of age. Animal protocols received the approval of the Institutional Animal Care and Use Committee of Huazhong University of Science and Technology. The methods were carried out in accordance with the approved guidelines.

### Isolation and identification of MLVECs

MLVECs were isolated according to the literature with slight modifications^23^. Mice were anesthetized, and then 50 units of heparin were injected via the jugular vein. An incision was made into the chest to insert a pulmonary artery catheter, and blood in the *vasa publica* was removed by perfusion with PBS. The edge of the lung tissues was excised and cut into 1-mm^3^ small pieces, which were placed in 60-mm petri dishes containing culture medium (MEM D-Val medium containing 2 mM glutamine, 10% FBS, 5% human serum, 50 μg/ml penicillin/streptomycin, 5 μg/ml heparin, 1 μg/ml hydrocortisone, 80 μg/ml endothelial cell growth supplement from bovine brain, 5 μg/ml amphotericin, and 5 μg/ml mycoplasma removal agent). After 60 h of incubation at 37 °C in 5% CO_2_, tissue blocks were removed, and adherent cells were continuously cultured for another 3 d. Endothelial cells were then purified using biotin-conjugated rat anti-mouse CD31 (PECAM-1) monoclonal antibody, BD IMag Streptavidin Particles Plus-DM, and an immunomagnetic separation system (BD Biosciences Pharmingen, San Diego, CA). The purified 3D cell culture was identified by cell morphology, Dil-Ac-LDL uptake and factor VIII staining before passaging. Generations 3–5 of the identified cells were used for experiments.

### Immunoblotting and immunoprecipitation

Cells were lysed in RIPA buffer supplemented with 1 mMol PMSF, 1 mMol Na_4_VO_3_ and protease inhibitor cocktail. The lysates were sonicated and centrifuged at 10,000 g for 10 min at 4 °C. The protein concentrations were measured with bicinchoninic acid kits. The samples were loaded equally for PAGE and then transferred onto nitrocellulose membranes. After blocking with 5% non-fat milk in TBST, the membranes were probed with primary antibodies for 2 h at room temperature or overnight at 4 °C and then incubated with HRP-conjugated secondary antibodies at room temperature for 1 h. The bands were determined using ECL reagent and quantified using ImageJ software (NIH).

For immunoprecipitation, the cell lysates were pre-cleared with 1 μg of normal IgG and 20 μl Protein A+G Agarose beads for 2 h at 4 °C. After centrifugation at 1000 g for 5 min, the supernatants were transferred to new tubes and incubated with 40 μl of Protein A+G Agarose beads and anti-Cav-1 or anti-RAGE antibodies overnight at 4 °C. The beads were collected for immunoblotting after 3 washes with PBS.

### Immunofluorescence

Cells plated on coverslips were fixed with 2% paraformaldehyde for 15 min and then washed 3 times with 100 mM glycine in HBSS for 10 min and once with HBSS for 10 min. After permeabilization with 0.1% Triton X-100 in HBSS for 30 min, the cells were incubated with primary antibodies (diluted with HBSS containing 5% horse serum and 0.2% BSA at a 1:200 dilution) overnight at 4 °C. Three washes with HBSS were followed by incubation with fluorescence-conjugated secondary Abs (1:200) for 1 h. After another 3 washes with HBSS, the cells were mounted on glass slides using Prolong Gold antifade reagents (Molecular Probes). Images were acquired with a confocal microscope (Zeiss LSM 510 Meta).

### Knockdown

A total of 5 × 10^5^ MLVECs were plated in 6-well plates and cultured overnight. A pool of three target-specific 20–25 nt siRNAs was used to knock down TLR2, TLR4, RAGE, clathrin or Cav-1 at a concentration of 25–50 nmol/l according to the protocol of Dharmacon. Forty-eight hours after transfection, the depletion of the target protein was confirmed by immunoblotting, and the cells were used in subsequent experiments.

### Electrotransfection

Transient transfection of MLVECs was performed according to the manufacturer’s instructions (Lonza). Briefly, 5 × 10^5^ MLVECs were resuspended in 100 μl of nucleofector solution mixed with 2 μg cDNA. Nucleofection was performed using programme M-003. Then, the cells were rapidly transferred to pre-equilibrated culture medium and incubated for 24–96 h at 37 °C. The viability of the cells was measured by vital dye exclusion. Successful transfection was confirmed by immunoblotting.

### Endocytosis assay with ^125^I-albumin

Endocytosis of ^125^I-albumin was assayed based on protocols from the literature^14^,^30^. Six-well plates were used to culture MLVECs until the cells formed confluent monolayers. HBSS containing unlabelled albumin was added at final concentrations of 0.1 mg/mL (in four wells) and 100 mg/mL (in two wells). Then, ^125^I-albumin (1 × 10^6^ cpm) was added to each well, and the plates were incubated for 30 min at 37 °C. The cells were washed successively with ice-cold acetate buffer (0.5 M NaCl and 0.2 M acetate, pH 2.5) and HBSS thrice to remove cell surface-bound ^125^I-albumin. The cells were lysed with 1 mL of Tris-HCl buffer (0.05 M Tris-HCl, 1% Triton X-100 and 0.5% SDS, pH 7.4), and the radioactivity of the cell lysates was detected using a γ counter (PerkinElmer, Inc.). The intake of specific ^125^I-albumin was calculated as total radioactivity (detection value of a sample containing 0.1 mg/mL unlabelled albumin) minus non-specific cell-related activity (detection value of a sample containing 100 mg/mL unlabelled albumin) and then corrected by the total cellular protein and expressed as cpm/mg cellular protein.

### Endocytosis assay of fluorescently labelled albumin

MLVECs were grown on cover slips until the cells formed confluent monolayers. AlexaFluor 488-labeled BSA (50 μg/mL) and unlabelled albumin (500 μg/mL) were added to the cell culture, and the culture was then incubated at 37 °C for 30 min. The cells were washed thrice with HBSS and examined under a confocal microscope (Zeiss LSM 510 Meta) to quantify the amount of fluorescence-labelled albumin endocytosed^14^,^30^.

### Transendothelial permeability assay with ^125^I-albumin

MLVECs were grown on fibronectin-coated microporous polyester Transwell membranes (12 wells, 1 cm^2^ growth area, 0.4 μm pore size; Corning Costar, Cambridge, MA) until the cells formed confluent monolayers. The upper chamber was supplemented with 0.5 mL of HBSS containing ^125^I-albumin (1 × 10^6^ cpm) and unlabelled albumin (0.1 or 100 mg/mL). The lower chamber was supplemented with 1.5 mL of HBSS containing the same amount of unlabelled albumin used for the upper chamber. The reaction time was 1 h, after which 200 μL of liquid sample was removed from the lower chamber and used for a radioactivity assay with a γ counter (PerkinElmer Inc.). The transendothelial permeability to ^125^I-albumin was calculated and expressed as μL/min.cm^2^ in accordance with the literature^14^,^30^.

### Transepithelial electrical resistance (TER) detection

TER detection was performed in accordance to the previous literature^14^,^31^. MLVECs were grown in culture plates containing gold-plated microelectrodes until the cells formed confluent monolayers. The positive and negative poles were connected to a synchronous phase sensitive amplifier. Computerized recording of the data related to voltage changes in HMGB1-treated cells was performed, and the data were expressed as the relative value to the initial reading of zero.

### Detection of permeability-surface area product (PS)

Mouse lungs were prepared as described previously^32^. In brief, mice were anesthetized, intubated, and ventilated. For pulmonary artery catheterization, the chests of the mice were surgically dissected, followed by perfusion with a modified Krebs-Henseleit solution at flow rate of 2 mL/min, a venous pressure of 3 cm H_2_O, and a pulmonary artery pressure of 8 ± 2 cm H_2_O.

Albumin PS was determined as previously described^14^ with slight modification. In brief, lung artery lavage was performed with a fluid containing 80000 counts/mL ^125^I-albumin and 0 or 100 ng/mL HMGB1. After 30 min, the lungs were irrigated with Krebs solution (containing 5% unlabelled albumin) for 6 min to remove excess ^125^I-albumin on the cell surface and within the cycling. Then, the attached tissues were quickly removed from the lung. The sample was weighed and used for γ radioactivity measurement. The PS value was calculated with the formula A/(Cp · t), where A and Cp are concentrations of tracer albumin in the tissue (in counts/g) and in the perfusate (in counts/mL), respectively, and t is the perfusion time for tracer albumin (30 min). The PS product was expressed as μL/(min·g) dry lung.

### Determination of lung tissue wet-dry weight ratio (W/D)

The wet lungs were weighed and then oven-dried at 60 °C for 72 h. The final weight was measured to calculate the W/D weight ratio.

### Statistical Analysis

The data are expressed as the mean ± SEM. One-way ANOVA with post-tests for individual treatments and Student’s Newman-Keuls test for post hoc comparisons were used to determine differences. Differences were considered significant when *p* < 0.05.

## Additional Information

**How to cite this article**: Shang, D. *et al.* High Mobility Group Box Protein 1 Boosts Endothelial Albumin Transcytosis through the RAGE/Src/Caveolin-1 Pathway. *Sci. Rep.* **6**, 32180; doi: 10.1038/srep32180 (2016).
